# Antibiotic Resistance of *Helicobacter pylori* in Patients with Peptic Ulcer

**DOI:** 10.3390/medicina59010006

**Published:** 2022-12-20

**Authors:** Thanh Binh Vu, Thi Nhu Quynh Tran, Thi Quynh Anh Tran, Dinh Luong Vu, Van Thuan Hoang

**Affiliations:** 1 Thai Binh University of Medicine and Pharmacy, Thai Binh 410000, Vietnam; 2 Thai Binh Department of Health, Thai Binh 410000, Vietnam

**Keywords:** *H. pylori*, antibiotic resistance, peptic ulcer

## Abstract

*Background and Objectives*: To determine the antibiotic resistance rate of *H. pylori* among patients with peptic ulcer. *Materials and Methods*: A cross-sectional monocentric study was conducted from January to December 2021 among patients aged from 16 years with gastrointestinal symptoms and esophagogastroduodenoscopy. Gastric mucosa biopsies were collected at the edges of the ulcer or at lesion sites for *H. pylori* culture. Five antibiotics (amoxicillin (AMX), clarithromycin (CLR), metronidazole (MTZ), levofloxacin (LEV), and tetracycline (TET)) were selected for antibiotic susceptibility testing. *Results*: One hundred and twenty-five patients were included, and the sex ratio was 0.6. Their mean age was 47.3 ± 14.2 years. All of the participants had gastritis, and 24.0% had duodenitis. A total of 21.6% of patients had a duodenal ulcer, and 12.8% had an antral ulcer. A total of 40 specimens have grown in *H. pylori* culture. The proportion of resistance to AMX, CLR, MTZ, LEV, and TET was 27.5%, 50%, 67.5%, 35%, and 5%, respectively. The proportion of multidrug resistance was 22.5%. The proportion of double resistance to AMX + CLR was 20.0%, AMX + MTZ was 15.0%, AMX + LEV was 2.5%, CLR + MTZ was 32.5%, and TET + MTZ was 5.0%. *Conclusions*: Our research results show that the treatment with MTX-TET or LVX-AMOX has the highest sensitivity rate. Therefore, practitioners should refer to these regimes to eradicate *H. pylori* in patients with gastric and duodenal ulcers. The reports on *H. pylori* eradication from different geographic areas show heterogeneous results. Therefore, continuous monitoring of antibiotic resistance of *H. pylori* in each population is very important. Having evidence helps clinicians to treat patients most effectively, reduce treatment costs, and limit the rate of antibiotic resistance.

## 1. Introduction

*Helicobacter pylori* is a Gram-negative bacterium that predominantly colonizes the stomach epithelium. It is characterized by a spiral shape, catalase, urease, and oxidase positive. It also has three to five polar flagella for motility. One of the most remarkable features of *H. pylori* is its ability to persist in the harsh environment of the stomach by metabolizing urea to ammonia via urease. This creates a neutral environment that envelops the bacterium [1].

*H. pylori* is a common cause of chronic gastritis, with more than half of the population worldwide suffering from it. The prevalence of *H. pylori* infection ranges from 40% to 90% depending on geographical region, economic conditions, and racial and ethnic groups [2]. The incidence of *H. pylori* infection is higher in low- and middle-income countries than in developed countries. In addition, the infection among ethnic groups in the same country is also different [2]. *H. pylori* infection causes chronic inflammation and potentially increases the risk of peptic ulcer and stomach cancer. *H. pylori* infection is the highest known risk factor for gastric cancer—the fifth most common cancer and the fourth leading cause of cancer death worldwide in 2020 [3]. Indeed, gastric cancer is considered to be the consequence of a multifactorial process involving bacterial virulence, host response, diet, and environmental factors [1]. *H. pylori* bacteria directly induce inflammation, converting normal mucosa into chronic superficial gastritis. This inflammation can progress to atrophic gastritis, then to intestinal metaplasia, and finally to dysplasia and cancer [1,3].

The World Health Organization (WHO) has identified that *H. pylori* eradication is one of the main measures to prevent stomach cancer [4]. Current regimens commonly used to treat *H. pylori* include proton pump inhibitors and a combination of two different antibiotics among the antibiotics amoxicillin (AMX), clarithromycin (CLR), metronidazole (MTZ), levofloxacin (LEV), and tetracycline (TET) [5]. However, like other bacteria, *H. pylori* have become increasingly resistant to antibiotics [6,7]. Antibiotic resistance of *H. pylori* is a major factor affecting the effectiveness of current treatment regimens. The determination of *H. pylori* susceptibility to antibiotics is necessary not only to monitor the epidemiological trend of bacteria to antibiotic resistance but also to select right regimen for patient care. This helps to improve the effectiveness of treatment, restrict adverse effects, and reduce the ability of bacteria to select for drug resistance.

Vietnam is a middle-income country located in an area with a high prevalence of *H. pylori*. There is an estimated population of nearly 100 million persons, distributed among 54 ethnic groups of different cultures, and over 60% of the population lives in rural areas. Previous studies in both hospitals and the community showed a high prevalence of *H. pylori* infection in Vietnam [8,9,10]. In addition, the rate of antibiotic resistance of *H. pylori* in the country is on the increase and dissimilar across geographical regions [11]. Therefore, the monitoring of *H. pylori* susceptibility in different regions helps clinicians to choose the optimal and effective initial treatment for patients. Therefore, we carried out this study in Thai Binh, a province located in the Red River Delta region of northeastern Vietnam, to determine the antibiotic resistance rate of *H. pylori* among patients with peptic ulcer.

## 2. Materials and Methods

### 2.1. Study Design and Participants

This is a cross-sectional monocentric study conducted at Thai Binh Medical University Hospital from January 2021 to December 2021.

Criteria for selection: All the patients aged from 16 years, regardless of geographical region and gender, with gastrointestinal symptoms and indication for esophagogastroduodenoscopy (EGD) and CLO positive test were selected in the study. Upon inclusion, participants were informed about the aims of the study, the unit in which the study was carried out, and how the collected data would be stored. They did not receive any incentives. They were also informed that this study did not use personal identifiers and that the collected data were used for research purposes only. They absolutely had the right to refuse or voluntarily participate in the survey. Their choice to participate in the study or not did not affect their use of health services. All participants provided their written informed consent.

We excluded the following patients: taking antibiotics or bismuth for four weeks or PPIs two weeks before EGD; currently pregnant or breastfeeding; and having a history of surgery in the esophagus, stomach, duodenum, or platelets 100,000/mm^3^ (because of the risk of bleeding during biopsies during endoscopy).

### 2.2. Esophagogastroduodenoscopy and Biopsies

EGD was performed in the Endoscopy service of the Thai Binh University of Medicine Hospital. During the EGD, four gastric mucosa biopsies were collected at the edges of the ulcer or at sites with suspected *H. pylori* lesions (slipping, lumpy mucosa). One biopsy piece was taken at the gastric body, and another at the gastric antrum was placed in a transportation medium for *H. pylori* culture. They were immediately transferred to the microbiology laboratory within 15 min. The two remaining biopsied pieces were taken for the urease test (HAMESCO Vietnam Company Limited, Hanoi, Vietnam) and histopathological examination.

### 2.3. H. pylori Culture and Antibiogram

Biopsy fragments taken for *H. pylori* culture were added to 500 µL of transportation medium containing 30% Glycerol. Then, they were ground in a culture medium (100 µL of Brain Heart Infusion (BHI) solution supplemented with 10% fetal bovine serum (FBS)). The fragments were cultured on an agar plate containing 1% isoVitale, 10% lysed sheep blood, a skin antibiotic mixture (vancomycin and trimethoprim), and amphotericin B (Nam Khoa Biotek Co., Ltd., Ho Chi Minh City, Vietnam). The agar plates were incubated at 37 °C in a specific microaerobic atmosphere (mixture of 5% O_2_, 10% CO_2,_ and 85%N_2_ gas) for three to seven days. A single colony in a culture medium was determined based on colony morphology and the features of *H. pylori* bacteria, including an S-shaped bacterium; Gram-negative; and being oxidase-positive, urease-positive, and catalase-positive.

According to the British Association of Chemical and Antibiotic Treatment [12] and the guideline of the Vietnamese Ministry of Health for use in *H. pylori* eradication among adults [13], we selected 5 types of antibiotics: amoxicillin (AMX), clarithromycin (CLR), metronidazole (MTZ), levofloxacin (LEV), and tetracycline (TET). This study also evaluated dual resistance with AMX–CLR, AMX–MTZ, AMX–LEV, CLR–MTZ, and MTZ–TET to help clinicians see the risk of failure in eradicating *H. pylori* when choosing the corresponding empiric regimen. Multidrug resistance was defined as the strain being resistant to three or more antibiotics [14,15].

Minimal inhibitory concentrations (MICs) of these five different antibiotics were determined by E-test (BioMerieux, Marcy-l’Étoile, France). According to the 2018 standards of the European Committee on Antimicrobial Susceptibility (EUCAST) to evaluate susceptibility, the resistance cutoff values were 0.125 µg/mL for AMX, 0.5 µg/mL for CLR, 8 µg/mL for MTZ and 1 µg/mL for LEV and TET [16].

### 2.4. Statistically Analysis

All statistical analyzes were performed using STATA software version 16.0 (SPSS Inc., Chicago, IL, USA). Qualitative variables were presented as frequency and percentage. Quantitative variables were presented as mean and standard deviation.

## 3. Results

### 3.1. Characteristics of Participants and Gastroduodenal Lesions

A total of 125 patients with peptic ulcer disease and having CLO positive test were included in the study, of whom 46 were male (36.8%), and 79 were female (63.2%). Hence, the sex ratio was 0.6. The mean age of participants was 47.3 ± 14.2 years (rang = 17–78 years) (Table 1).

The EGD showed that all of the participants had gastritis, and 24.0% (30/125) had duodenitis. In addition, 21.6% (27/125) patients had a duodenal ulcer, and 12.8% (16/125) had an antral ulcer (Table 1).

Histopathological results showed that chronic inflammation was the most frequent, followed by moderate/severe inflammation, atrophic gastritis, dysplasia, and metaplasia (Table 1).

### 3.2. H. pylori Culture and Antibiotic Resistance

Biopsy fragments of 125 patients were taken for *H. pylori,* but only 40 (32.0%) specimens were culture-positive.

The antibiogram showed that the proportion of resistance to AMX, CLR, MTZ, LEV, and TET was 27.5% (11/40), 50% (20/40), 67.5% (27/40), 35% (14/40), and 5% (2/40), respectively.

Out of 40 *H. pylori* strains, three (7.5%) were sensitive to all five antibiotics; 11 (27.5%) were mono-resistant, 42.5% (17/40) were dual-resistant, 17.5% (7/40) were triple-resistant, 5% (2/40) were quadruple resistance, and no strain was resistant to all of five tested antibiotics. Hence, the proportion of multidrug resistance was 22.5% (9/40).

**Figure 1:**
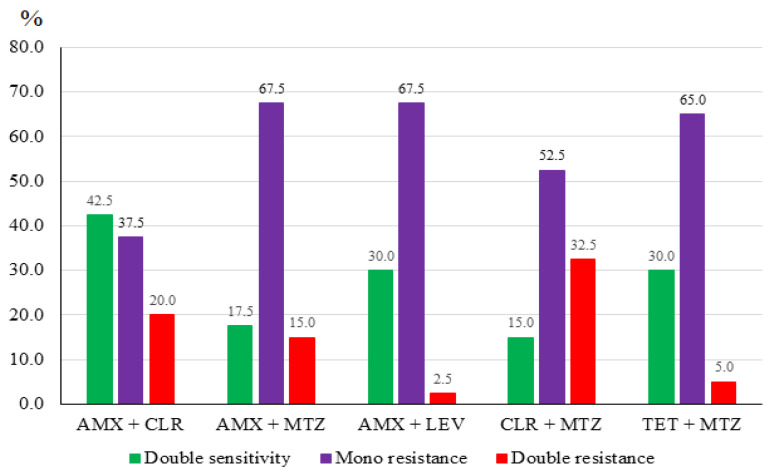
Frequency of susceptibility and resistance to antibiotics of *H. pylori* strains (amoxicillin (AMX), clarithromycin (CLR), metronidazole (MTZ), levofloxacin (LEV), and tetracycline (TET)).

Analysis of antibiotic sensitivity in *H. pylori* bacteria to select an effective eradication therapy found that double resistance to AMX + CLR was 20.0% (8/40), and susceptibility to both was 42.5% (17/40); double resistance to AMX + MTZ was 15.0% (6/40), and susceptibility to both was 17.5% (7/40); double resistance to AMX + LEV was 2.5% (1/40), and susceptibility to both was 30.0% (12/40); double resistance to CLR + MTZ was 32.5% (13/40), and susceptibility to both was 15.0% (6/40); double resistance to TET + MTZ was 5.0% (2/40), and susceptibility to both was 30.0% (12/40) (Figure 1).

## 4. Discussion

In our study, 125 patients with CLO-positive tests were enrolled, but only 40 (32.0%) were positive in culture. *H. pylori* is known to be difficult to grow and requires specialized culture media [17]. The positive rate depends on the bacterial concentration and the culture medium used. In addition, identification based on colony morphology and characteristics of this bacterium lacks specificity; therefore, they could be excluded from the analysis. Furthermore, *H. pylori*-positive culture was found to be dependent on the type of pathology involved, and the number of biopsies taken could also affect the sensitivity of the culture [18]. A single biopsy specimen from the antrum provides high sensitivity, but it is not sufficient for a reliable diagnosis. In this study, we took two biopsies (one from the gastric body and another from the antrum) and mixed them before culturing. Indeed, *H. pylori* can be distributed in patches, and the chance of detecting the organism increases while more biopsies are analyzed [17]. However, the difference in ecosystem conditions and type of microvilli between the antrum and gastric body could be the decisive factor affecting the adaptation of the different *H. pylori* strains found. Therefore, the separate culture of biopsies should also be discussed in situations where the patient has multiple lesions.

Another explanation for the low rate of positive cultures for *H. pylori* is urease produced by other bacteria. Indeed, the enzyme urease is produced by several taxonomically miscellaneous bacterial species, including normal flora [17]. This fact might be responsible for the large number of CLO-positive but culture-negative samples.

Vietnam is an area with a high prevalence of *H. pylori* infection in the community, from 75 to 90%, and an average rate of stomach cancer with an incidence rate of 18.4 cases/100,000 habitats [19]. Peptic ulcer also accounts for a relatively high proportion of gastrointestinal diseases [8]. Since being discovered by Marshall and Warren, *H. pylori* bacteria has been an infectious agent that has been shown to be related to lymphoma, gastric cancer, iron deficiency anemia, and thrombocytopenia bleeding [20]. *H. pylori* infection is one of the most common chronic bacterial infections. *H. pylori* eradication plays an important role in the treatment of peptic ulcer disease. Therefore, a successful eradication therapy of this agent is very important to reduce the risk of developing gastric cancer [21]. Unfortunately, our study showed that antibiotic-resistant *H. pylori* strains were common in the Vietnamese community. The standard OAC regimen (Omeprazole + AMX + CLR) has been recorded to have a success rate of 90–95%, but currently, the effectiveness of this regimen has been significantly reduced [22]. In a recent randomized clinical trial study including 369 *H. pylori*-infected patients, the eradication rate in the 7-day and 14-day treatment was 64.0% and 66.0%, respectively [22]. Indeed, the rate of antibiotic resistance of *H. pylori* is increasing and varies by region and over time [5].

Previous studies have found that the prevalence of antibiotic-resistant *H. pylori* is increasing in Vietnam, especially for triple therapy, which was once the first-line therapy in eradicating *H. pylori* [21,23,24]. There are many reasons leading to antibiotic resistance of *H. pylori* bacteria, but the non-adherence to treatment, inadequate length of therapy, overuse of antibiotics, and rapid adaptation of *H. pylori* to therapeutic drugs mainly account for treatment failure [25]. In addition, the instructions on how to take the drug are not clear and specific, causing the patient not to take the drug correctly and resulting in treatment failure even though the regimen is still sensitive.

In our study, the proportions of resistance to AMX, CLR, MTZ, LEV, and TET were 27.5%, 50%, 67.5%, 35%, and 5%, respectively. In addition, 7.5% were sensitive to all five antibiotics, 27.5% were mono-resistant, 42.5% were dual-resistant, 17.5% were triple-resistant, and 5% were quadruple-resistant. No strain was resistant to all five tested antibiotics. Our results are similar to other studies in Vietnam and around the world carried out in recent years [14,15,21,25,26]. However, the rate of antibiotic resistance is higher than in another study conducted in Vietnam in 2008. Among the 103 *H. pylori* strains isolated from patients in Ha Noi et Ho Chi Minh city, the resistance rates were 0% (AMX), 33% (CLR), 69.9% (MTZ), 18.4% (LEV), and 5.8% (TET) [21].

Our study shows the high resistance rate for CLR and MTZ, which are recommended as first-line therapies in Asian countries [27]. In addition, 15% of *H. pylori* strains are resistant to all these antibiotics. Particularly, CLR resistance has increased rapidly in many countries over the past decade, with the proportion ranging from 15% to 50% [28,29,30,31]. This is the main cause leading to the failure of standard regimens for *H. pylori* treatment. In a Vietnamese study, the eradication success did not differ in age, sex, and family membership, but there were significantly more failures in the CLR regimen [25]. The previous studies confirmed that macrolide resistance could decrease by 70% of the effectiveness of treatment [32]. A meta-analysis showed that CLR resistance in triple therapy consisting of PPI, AMX, and CLR decreased treatment efficacy by 66% [33].

In our study, the resistance rate of *H. pylori* to MTZ was higher than that of previous studies [15,32,34,35]. MTZ resistance ranged from 20% to 40% in Europe and United States. In Canada, this proportion was reported from 18% to 22% [34]. This can be explained by the higher rate of MTZ overuse in the Vietnamese community [35]. Indeed, individuals can buy antibiotics themselves without a medical prescription. MTZ is commonly used to treat not only *H. pylori* infection but also other infections, including gastrointestinal infections and periodontal and gynecological diseases [32]. The rate of MTZ resistance has increased in recent times. This is a major factor leading to the reduction in the effectiveness of standard triple therapy in most countries worldwide. Therefore, CLR-MTZ would not be used as a first-line regimen for *H. pylori* treatment.

Recently, LEV has been prescribed as an alternative drug to eliminate *H. pylori* infection in patients after failure of first-line therapy [36,37]. However, rates of LEV resistance appear to be increasing, and it might reduce the effectiveness of treatment with the AMX-LEV regimen [38,39]. In the present study, we showed a high rate of resistance to LEV, higher than AMX. This could be explained by the increasing use of LEV in cases of respiratory tract infections. In addition, other fluoroquinolones, such as nalidixic acid, ciprofloxacin, and ofloxacin, which were commonly used in Vietnam, may lead to cross-resistance with LEV [21]. On the other hand, TET resistance was relatively low (5.0%). This result is consistent with that reported in previous studies [7,21,27,40]. TET was not commonly used in Vietnam. Therefore, the TET-based regime could be a useful alternative in Vietnam, as recommended in the guidelines [7,27].

Our study has some limitations. Firstly, this is a single-center study with a small sample size and a relatively low growth rate of *H. pylori* culture. Therefore, the study results may not be representative of the Vietnamese population. On the other hand, the study of antibiotic resistance of *H. pylori* through culture and antibiogram also only indirectly reflects the effectiveness of treatment. We did not re-evaluate patients for clinical success rates. In addition, the use of culture media containing antibiotics could lead to the selection of strains with high adaptability and thus increase antibiotic resistance rate. An antibiotic-free culture medium should be investigated for use in monitoring the growth kinetics. It could explain the metabolic processes that allow the capture and use of nutrients from the culture medium and evaluate the true rate of antibiotic resistance of *H. pylori*. Furthermore, we evaluated only qualitative values of resistance without considering bacteriostasis, which must be considered in resistance phenomena in reality. In fact, it is the first perceptible phenomenon in resistance, depending on the adaptive processes and mutations of *H. pylori* [41]. The most recent data available from the literature showed different resistance mechanisms of this bacterium, including membrane permeability pump efflux systems, redox intracellular potential, and point mutations. Molecular markers assessment is essential to identify particularly virulent strains and pathovars precisely because of the differential emergence of antibiotic resistance mechanisms and genes. However, due to the lack of facilities, molecular biology techniques could not be performed in our hospital.

## 5. Conclusions

Our research results show that the treatment with MTX-TET or LVX-AMOX has the highest sensitivity rate. Therefore, practitioners should refer to these regimes to eradicate *H. pylori* in patients with gastric and duodenal ulcers. However, the reports on *H. pylori* eradication from different geographic areas show heterogeneous results. One treatment regimen may be effective in one area but not in another. Therefore, continuous monitoring of antibiotic resistance of *H. pylori* in each population is very important. It helps clinicians have evidence for treating patients most effectively, reducing treatment costs, and limiting the rate of antibiotic resistance. The mechanisms of antibiotic resistance of *H. pylori* also need to be further investigated, and consideration should be given to the introduction of drug-resistance inhibitors together with corresponding antibiotics. The indications for the dosage of the antibiotics should be associated with previous data on the susceptibility profiles of the isolated bacteria, including molecular characterization data. This will allow obtaining useful information for more efficient treatment and reduce the potential emergence of resistance. In addition, monitoring of water resources, especially in urban areas where high concentrations of antibiotics are combined with a variety of *H. pylori* strains, is also important to limit the incidence of antibiotic-resistant *H. pylori*.

## Figures and Tables

**Table 1:** Characteristics of studied population.

Characteristics	*n* = 125 *n*	%
Gender		
Male	46	36.8
Female	79	63.2
Age (years)		
Mean ± SD	47.3 ± 14.2	
Range	17–78	
Endoscopy findings		
Gastritis	125	100
Duodenitis	30	24.0
Gastric ulcer	16	12.8
Duodenal ulcer	27	21.6
Histopathology findings		
Chronic inflammation	104	83.2
Moderate/severe inflammation	83	66.4
Atrophic gastritis	65	52.0
Metaplasia	12	9.6
Dysplasia	20	16.0

## Data Availability

The data presented in this study are available on request from the corresponding author [T.N.Q.T or V.T.H] upon reasonable request.
